# P08 The difficulty of *Pseudomonas aeruginosa* eradication in a post-spinal anaesthesia meningitis

**DOI:** 10.1093/jacamr/dlac053.008

**Published:** 2022-05-31

**Authors:** Entela Kolovani, Ergys Ramosaço, Artur Xhumari, Nevila Kryemadhi, Arjan Harxhi, Najada Çomo

**Affiliations:** 1 Infectious Diseases Hospital, UHC “Mother Theresa”, Tirana, Albania; 2 Neurosurgery Clinic, UHC “Mother Theresa”, Tirana, Albania

## Abstract

**Background:**

Meningitis and ventriculitis due to *Pseudomonas aeruginosa* are uncommon. They are commonly secondary hospital-onset and typically related to neurosurgical procedure. Previous studies have shown *P. aeruginosa* to be responsible for 1%–18% of nosocomial meningitis cases. Both treatment failure and relapses are known to occur, and the recorded mortality is high, approaching 80% in some studies.

**Objectives:**

We report a case of *P. aeruginosa* meningitis in a 26-year-old woman following spinal anaesthesia for caesarean section, its clinical characteristics, treatment challenges in the era of high generation antibiotics and the outcomes.

**Results:**

A 26-year-old woman was hospitalized after 1 week of her third child delivery by caesarean section with spinal anaesthesia and 3 days after high fever and headache. Lumbar puncture was performed immediately, and leucocyte cell count was 723 cell/dL with 94% segmented neutrophils. CSF glucose was very low, and protein was 420 mg/dL. *P. aeruginosa* was isolated and it was susceptible to all antibiotics of standard panel. Cranial and lumbar region CT scans were normal. We modified empirical treatment and started ceftazidime and gentamicin. Three days after de-hospitalization in improved condition, she had high fever and strong headache and came back to hospital. We started treatment with imipenem and ciprofloxacin and 2 months after we performed several lumbar punctures, cranial MRIs and changed different antibiotic regimens with third generation cephalosporins, aminoglycosides, carbapenems and colistin; her clinical situation such as headache and fever and CSF did not show any sign of improvement. Susceptible *P. aeruginosa* was isolated each time we examined her CSF. She decided to leave the hospital and 2 weeks after her headache worsened and a convulsive attack happened. The cranial MRI showed hydrocephaly and a white precipitate (pus) in the left lateral ventricle. An extraventricular drain helped to resolve hydrocephaly and intraventricular colistin was injected until CSF was sterilized. After 1 month she was in a very good condition and a ventriculo-peritoneal shunt was placed.

**Conclusions:**

*P. aeruginosa* meningitis was associated with some treatment difficulties. Even after an early nosological and microbiological diagnosis and an appropriate choice of antibiotics, based on bacterial meningitis treatment criteria and antibiogram, we couldn't eradicate the bacteria from CSF. To achieve success in this nosocomial infection from a not careful asepsis, sometimes a prolonged antibiotic and local treatment with intraventricular or intrathecal therapy is needed.

